# Upbeat vertical nystagmus after brain stem cavernoma resection: a rare case of nucleus intercalatus/nucleus of roller injury

**DOI:** 10.1007/s00415-020-09891-4

**Published:** 2020-05-26

**Authors:** Torstein R. Meling, Aria Nouri, Adrien May, Nils Guinand, Maria Isabel Vargas, Christophe Destrieux

**Affiliations:** 1 Department of Neurosurgery, Geneva University Hospitals, University of Geneva, Rue Gabrielle-Perret-Gentil 4, 1205 Geneva, Switzerland; 2 Faculty of Medicine, University of Geneva, Geneva, Switzerland; 3 Division of Otorhinolaryngology Head and Neck Surgery, Geneva University Hospitals, Geneva, Switzerland; 4 Department of Diagnostic Neuroradiology, Geneva University Hospitals, Geneva, Switzerland; 5 UMR 1253, iBrain, Université de Tours, Inserm, Tours, France; 6 CHRU de Tours, Tours, France; 7 Institute of Clinical Medicine, Faculty of Medicine, University of Oslo, Oslo, Norway

**Keywords:** Cavernoma, Nucleus intercalatus, Nystagmus, Complication

## Abstract

**Introduction:**

CNS cavernomas are a type of raspberry-shaped vascular malformations that are typically asymptomatic, but can result in haemorrhage, neurological injury, and seizures. Here, we present a rare case of a brainstem cavernoma that was surgically resected whereafter an upbeat nystagmus presented postoperatively.

**Case report:**

A 42-year old man presented with sudden-onset nausea, vomiting, vertigo, blurred vision, marked imbalance and difficulty swallowing. Neurological evaluation showed bilateral ataxia, generalized hyperreflexia with left-sided predominance, predominantly horizontal gaze evoked nystagmus on right and left gaze, slight left labial asymmetry, uvula deviation to the right, and tongue deviation to the left. MRI demonstrated a 13-mm cavernoma with haemorrhage and oedema in the medulla oblongata. Surgery was performed via a minimal-invasive, midline approach. Complete excision was confirmed on postoperative MRI. The patient recovered well and became almost neurologically intact. However, he complained of mainly vertical oscillopsia. The videonystagmography revealed a new-onset spontaneous upbeat nystagmus in all gaze directions, not suppressed by fixation. An injury of the rarely described intercalatus nucleus/nucleus of Roller is thought to be the cause.

**Conclusion:**

Upbeat nystagmus can be related to several lesions of the brainstem, including the medial longitudinal fasciculus, the pons, and the dorsal medulla. To our knowledge, this is the first case of an iatrogenic lesion of the nucleus intercalatus/nucleus of Roller resulting in an upbeat vertical nystagmus. For neurologists, it is important to be aware of the function of this nucleus for assessment of clinical manifestations due to lesions within this region.

**Electronic supplementary material:**

The online version of this article (10.1007/s00415-020-09891-4) contains supplementary material, which is available to authorized users.

## Introduction

Central nervous system cavernomas are a type of blood vessel malformations affecting the veins, frequently described as raspberry shaped. They have an estimated prevalence ranging between 0.02 and 0.5% in the general population [1] and represent 5–15% of vascular malformations in the brain. Of these, 15–18% have been reported to represent brainstem cavernomas [2]. While typically benign and innocuous, they may become clinically important in the setting of bleeding, where they can lead to local ischemia, oedema, inflammation, irritation and seizures [1].

## Case report

A 42-year old man from Switzerland on vacation in Spain was air-lifted to our hospital after being hospitalized there for 1 week. He was initially presented with sudden-onset nausea, vomiting, vertigo, blurred vision, and marked imbalance. Additionally, the patient described difficulty swallowing. A head non-enhanced CT scan revealed a well-delineated, round, and hyperdense centimetric lesion in the medulla oblongata and a subsequent MRI demonstrated a 13-mm brainstem cavernoma with haemorrhage and oedema (Supplementary Fig. 1). Neurological evaluation showed bilateral ataxia, generalized hyperreflexia with left-sided predominance, a predominantly horizontal gaze evoked nystagmus on right and left gaze, slight left labial asymmetry, uvula deviation to the right, and tongue deviation to the left. Romberg sign was positive. No other neurological deficits were noted.

Due to the symptoms’ persistence and severity 2 weeks after the initial bleed, we proposed a microsurgical excision of the cavernoma. Surgery was performed under neuromonitoring for cranial nerves IX–XII, as well as sensory and motor-evoked potentials. A 10-cm midline skin incision and a 17-mm suboccipital craniectomy was undertaken. Intradurally, a subtonsillar approach exposed the floor of the 4th ventricle and spots of underlying haemorrhage (Supplementary Fig. 2). A complete excision of the cavernoma was executed without changes in the neuromonitoring recordings (Supplementary Fig. 3).

**Fig. 1:**
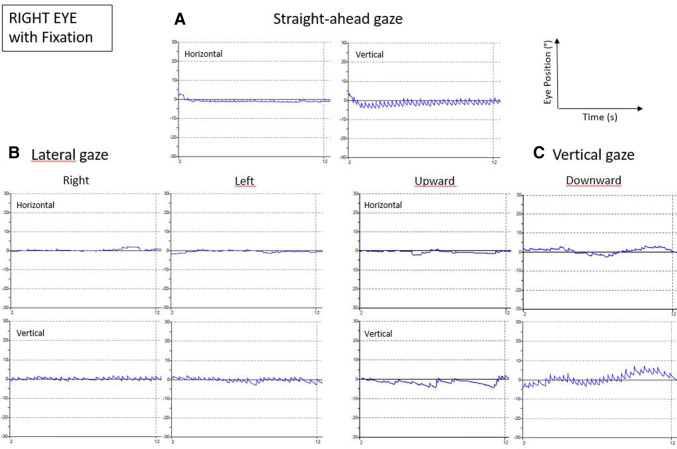
Video-oculographic recording of the horizontal and vertical position of the right eye is shown in different gaze conditions: **a** straight-ahead gaze, **b** lateral gaze and **c** vertical gaze. The upbeat component is present in all gaze conditions. In the straight-ahead and downward gaze conditions, a discrete horizontal component to the right is observed

Postoperatively, the hypoglossal improved markedly, but tongue paresis on the left was still present at 2 weeks, as was his labial asymmetry. His dysarthria, dysphagia and balance were subjectively better, but he complained of oscillopsia, mainly oriented in the vertical plane. On postoperative clinical examination, besides a still positive Romberg test, the patient showed a new-onset upbeat nystagmus (Video 1). A 2D-videonystagmography (Difra, Eupen, Belgium) showed a spontaneous upbeat nystagmus in all gaze directions, which was not suppressed by fixation (Fig. 1). Saccades were normal, horizontal and vertical smooth pursuit was impaired, gain of the optokinetic nystagmus was reduced. Video Head Impulse Test (Synapsys, Marseilles, France) was normal for all canals.

**Fig. 2:**
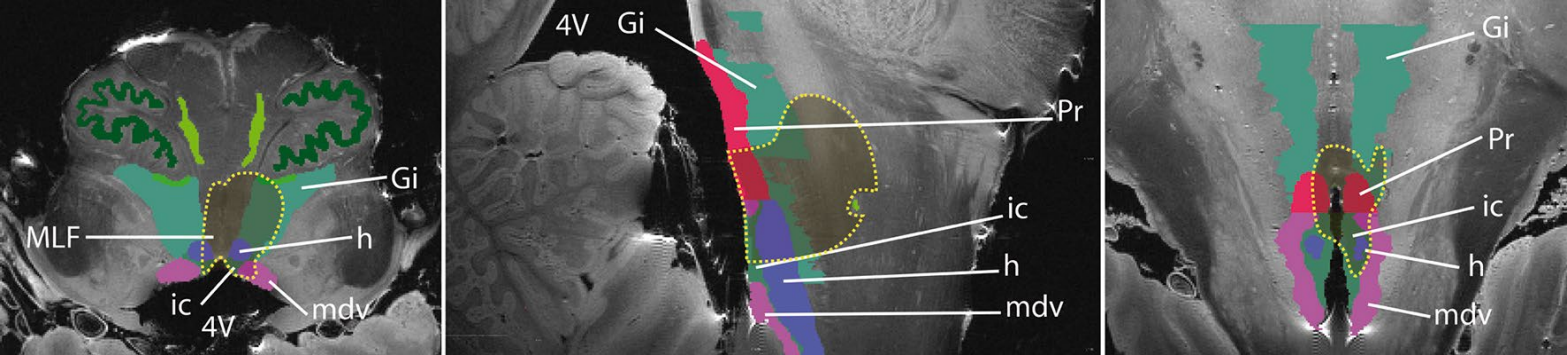
Ex vivo image of the brain stem obtained at 11.7 T (T2 weighted, 100 microns isotropic) showing the hypoglossal (h), motor dorsal of the vagus (mdv), giganto-cellular reticularis (Gi), prepositus (Pr) and intercalated (ic) nuclei and the middle longitudinal fasciculus in axial (**a**), sagittal (**b**), and coronal (**c**) orientations. The postoperative cavity was rigidly co-registered onto this atlas, displayed in brown and bordered by a yellow dot line [3]

To explore possible causes for this upbeat nystagmus, the postoperative T1 weighted MRI was rigidly co-registered (FLIRT) to a MRI atlas obtained at 11.7 T from an ex vivo specimen including 124 grey matter anatomical classes [3]. Limits of the postoperative cavity were displayed onto the atlas, showing a bilateral (left predominant) lesion of the rostral hypoglossal and caudal prepositus nuclei, intercalated nuclei and giganto-cellular reticular nuclei, and medial longitudinal fasciculi. A left partial lesion of the motor dorsal nucleus of the vagal nerve was also present (Fig. 2).

## Discussion

The patient reported here presented preoperatively with lower cranial nerve deficits and a horizontal spontaneous nystagmus. A postoperative videonystagmography revealed an upbeat nystagmus in all gaze directions, not suppressed by fixation (Fig. 1). On postoperative MRI, the resection cavity can be found on the dorso-medial side of the medulla oblongata, just caudal to the ponto-medullary junction and included several structures possibly related to vertical control of gaze, namely both caudal parts of middle longitudinal fasciculus (MLF), and the perihypoglossal nuclei, including the intercalated nucleus and possibly the nucleus of Roller (RON).

Gaze fixation in the vertical direction implies a balanced activity of muscles directing the gaze upwards (namely the superior rectus and inferior oblique muscles innervated by the oculomotor nerve) and muscles directing the gaze downwards (inferior rectus and superior oblique muscles, respectively, receiving afferents from the oculomotor and trochlear nerves). A permanent decreased activity of muscles promoting elevation relative to the ones for depression induces a slow downward movement periodically compensated by upward directed saccades. When such a nystagmus is present in the straight-ahead position as this is the case in the reported patient—it is referred as upbeat nystagmus [4]. At least two mechanisms control this gaze vertical balance:

First, direct connections exist between the vestibular complex and oculomotor nuclei through the MLF: the medial vestibular (excitatory) and superior vestibular (inhibitory) nuclei both give tracts controlling nuclei for eyeball elevation (oculomotor nucleus) and depression (oculomotor and trochlear nuclei). Lesion of the MLF, as observed in internuclear ophthalmoplegia, is usually associated with a vertical gaze-evoked nystagmus. Nevertheless, since both MLT conduct inhibitory and excitatory input for upward and downward directions, a bilateral MLF lesion does not induce nystagmus in the straight-ahead position of gaze [4]. It thus seems unlikely that the upbeat nystagmus, which was observed in all gaze—including straight-ahead—directions in the reported patient, was related to the bilateral MLF lesion.

**Fig. 3:**
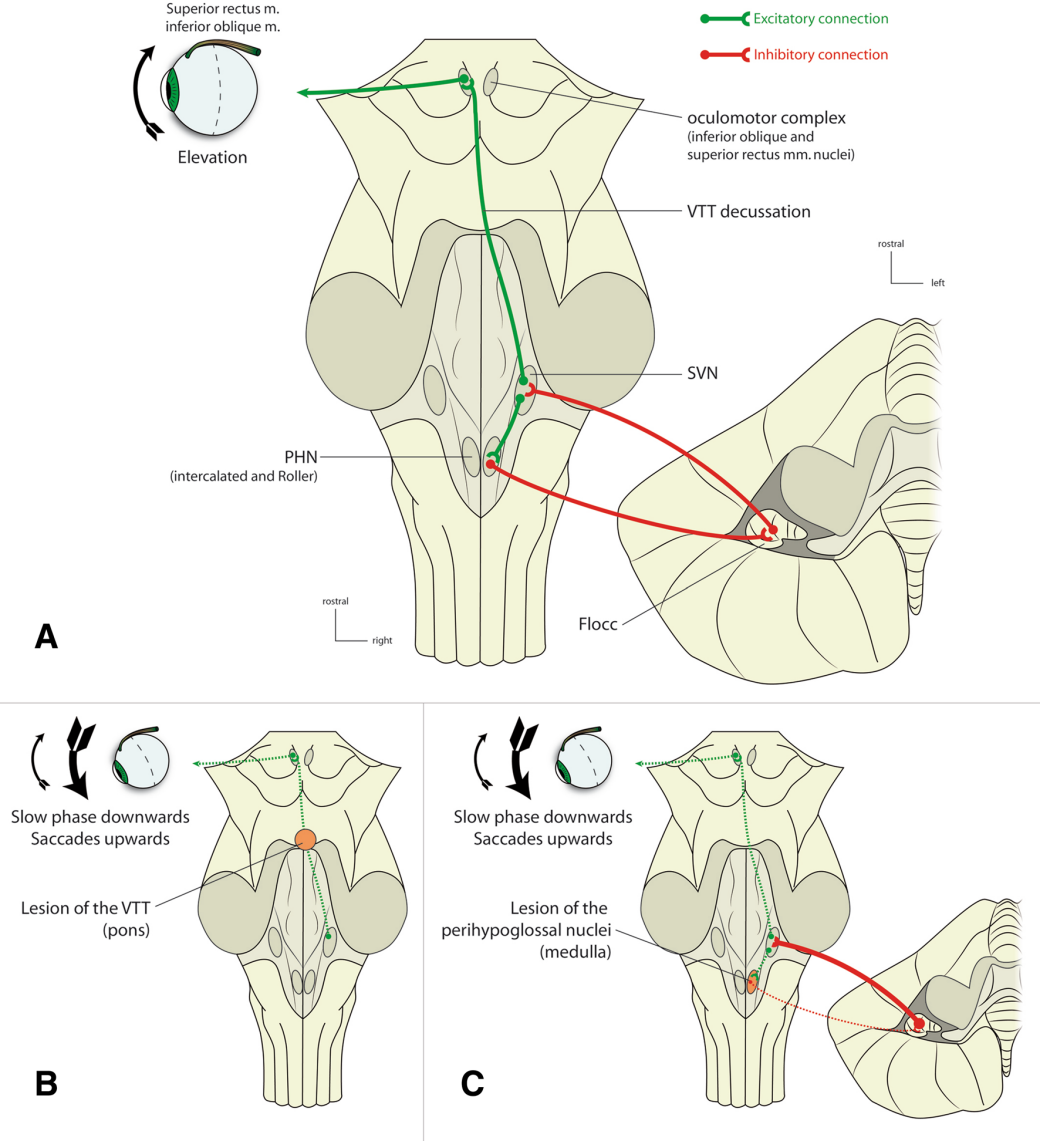
Pathophysiology of vertical nystagmus proposed by Deselligny and Milea [4]. **a** Normal situation. The ventral tegmental tract (VTT) originates from the superior vestibular nucleus (SNV), decussates at the pontine level and projects onto the nuclei of the inferior oblique and superior rectus muscles. It thus induces an elevation of the eyeball. The SVN is also excitatory for the perihypoglossal nuclei (PHN), which inhibit the flocculus (Floc). The latter finally sends back inhibitory projections to SVN. **b** Upbeat nystagmus secondary to lesion of the VTT (pons). Since the SVN-VTT complex cannot stimulate elevating muscles, a slow downward movement of the gaze occurs, periodically compensated by saccades directed upwards. **c** Upbeat nystagmus secondary to lesions of the PHN (medulla). A lesion of the intercalated or Roller nuclei suppress the physiological inhibition of the flocculus (Floc). This reinforces the normal inhibition of SVN by Floc. Finally, the decreased activity in SVN has the same consequences as lesion A (upbeat nystagmus)

 A second more complex circuitry connects the vestibular system to the oculomotor nuclei and was summarized by [4] (Fig. 3): (1) excitatory output from the superior vestibular nucleus (SVN) projects onto the superior rectus/inferior oblique nuclei via the ventral tegmental tract (VTT). The later originates from the SVN in the lateral tegmentum of the medulla, and ascends medially and ventrally to reach the medial limit of the medial lemniscus, at the junction between the tegmentum and the basilar part of the pons. It then decussates at the upper part of the reticulo-tegmental nucleus to follow the medial aspect of the contralateral medial lemniscus and ends in the superior rectus/inferior oblique nuclei [5]. Lesion of the VTT induces a decrease of elevating muscles excitatory input which results in a slow downward motion of the eyeball, periodically compensated by an upbeat nystagmus. This is reported in extensive bilateral damage to the ventral tegmentum and posterior part of the basis pontis [6–8] and, more interestingly, in focal lesions of the VTT decussation [5]. In our case, the postoperative cavity, located at the medial part of the tegmentum of the medulla, seems unlikely to involve the VTT, which lies more lateral at this level and becomes medial more rostrally. (2) The SVN, at the origin of this SVN–VTT pathway, is controlled by a loop involving some of the perihypoglossal nuclei (PHN), located within the dorsal medulla, and the flocculus: SVN sends excitatory projections to PHN, which inhibit the flocculus. The later finally projects back to inhibit the SVN. As a consequence, a PHN lesion suppresses inhibition of the flocculus, which increases its inhibitory action onto the SVN. This finally decreases the activity of the SVN–VTT pathway, leading to an upbeat nystagmus. The PHN include the prepositus nucleus, located at the rostral tip of the hypoglossal nucleus, the intercalated nucleus, and the *sublingual nucleus of Roller*, the two last ones being potential candidates for playing a role in the SVN–PHN–Flocculus SVN circuit:

- Staderinis intercalatus nucleus (SIN), formerly known as area lateralis of the hypoglossal trigone [9] is located between the hypoglossal nucleus and dorsal nucleus of the vagus nerve [10, 11]. Since the 1970s, it has been studied by mapping its afferent and efferent pathways primarily in cats and monkeys [12, 13]. Brodal [14, 15] demonstrated connections between the perihypoglossal nuclei and flocculus, paraflocculus and nodulus. Mergner et al. [16] described its afferent projections particularly from the medial and descending vestibular nuclei, as well as from the nucleus prepositus. Other authors studied the spinal afferents to the SIN and found it to be a complex relay of cervical and vestibular afferent information to premotor structures involved in both neck and ocular motor control [17]. In 1998, Hirose et al. [8] and Munro et al. [18] each reported one case of upbeat nystagmus due to unilateral medial medullary infarction involving the SIN and in 2010, Saito et al. presented a multiple sclerosis patient with a SIN lesion responsible for the primary position of upbeat nystagmus [19]. SIN is thus considered to participate to the vertical cerebello-vestibular integrator involved in stabilizing vertical gaze [8, 10, 18, 20].
- Another candidate which may be involved in the inhibition of the flocculus may be the nucleus of Roller (RON). This is a very small nucleus, located just ventral to the cranial tip of the hypoglossal nucleus [11], which gets strong projections form the SVN and projects to the flocculus [4]. The postoperative cavity included both SIN and probably both RON, despite the latter were not visible on ex vivo 11.7 T MRI due to their very small size (less than 1 mm^3^) and a lack of contrast to the surrounding structures. This bilateral lesion is consistent to the literature, since a unilateral lesion would also induce a torsional component in the nystagmus, was absent in the reported case [4].

This report entails many strengths and some limitation. We benefited from a well-described clinical examination combined with pre- and postoperative high-resolution MR imaging. Furthermore, we have documented the upbeat nystagmus with a video, videonystagmography, and correlated the lesion with ex vivo 11.7 T brainstem imaging. Six months after surgery, the videonystagmography was repeated and showed the persistence of the spontaneous upbeat nystagmus. Unfortunately, we do not have a pre-operative videonystagmography.

## Conclusion

To our knowledge, this is the first case of an iatrogenic SIN/RON lesion resulting in an upbeat vertical nystagmus. For the neurologists, it is important to be aware of the function of these nuclei for assessment of potential clinical manifestations due to lesions within this region. For the neurosurgeon, intimate knowledge of the brainstem anatomy is crucial to lower the morbidity associated with extirpation of brainstem cavernomas.

## Electronic supplementary material

Below is the link to the electronic supplementary material.

Supplementary file1 Fig. 1 Preoperative T2-weighed MRI images in axial (A) and sagittal (B) cuts. (JPG 2923 kb)

Supplementary file2 (JPG 2857 kb)

Supplementary file3 Fig. 2 Intraoperative view of the floor of the 4th ventricle. (JPG 1644 kb)

Supplementary file4 Fig. 3 Postoperative 3D T2-weighed high resolution MRI sequences showing complete resection of the cavernoma without radiological complications in axial (A) and sagittal (B) cuts. (JPG 3222 kb)

Supplementary file5 (JPG 3014 kb)

Supplementary file6 Video file 1. Postoperative video of patient showing a vertical nystagmus. Supplementary file 1. Fluid-attenuated inversion recovery (FLAIR) coronal sequences illustrating cavernoma before surgery (a, b) surrounded by oedema (white arrow in a) and high signal of the medial lemniscus (black arrow in a) and after surgery (b, c) showing the postoperative cavity with a decrease of mass effect (c) and high signal of the intercalatus nucleus (arrow in d). (MP4 21528 kb)
