# Supplementary figures and images for: Upbeat vertical nystagmus after brain stem cavernoma resection: a rare case of nucleus intercalatus/nucleus of roller injury
Source: J Neurol. 2020 May 26;267(10):2865–70. doi: 10.1007/s00415-020-09891-4 (PMC7501124; doi:10.1007/s00415-020-09891-4)

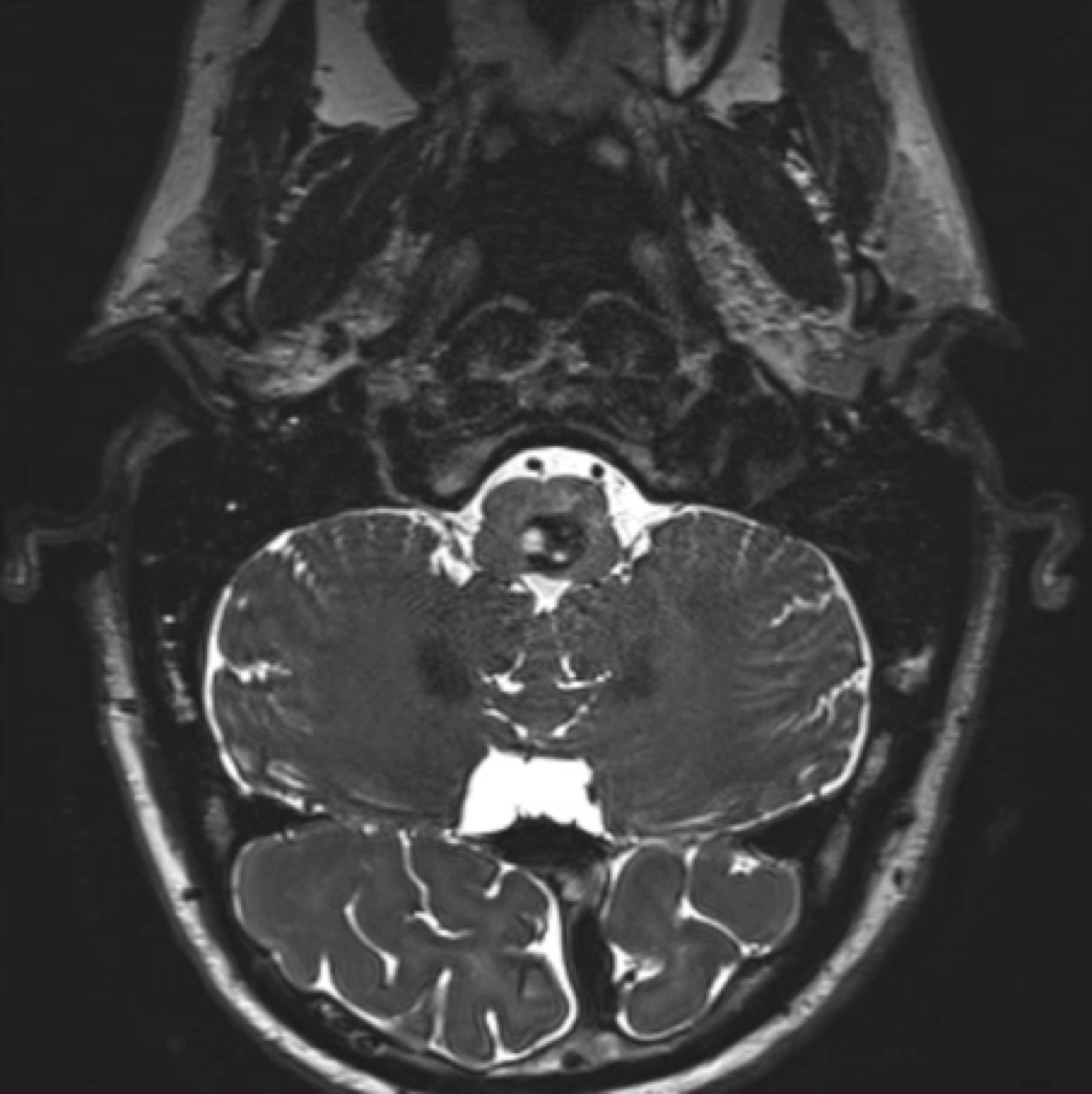

Supplement: Supplementary file 1 — Supplementary file1 Fig. 1 Preoperative T2-weighed MRI images in axial (A) and sagittal (B) cuts. (JPG 2923 kb) [file 415_2020_9891_MOESM1_ESM.jpg]

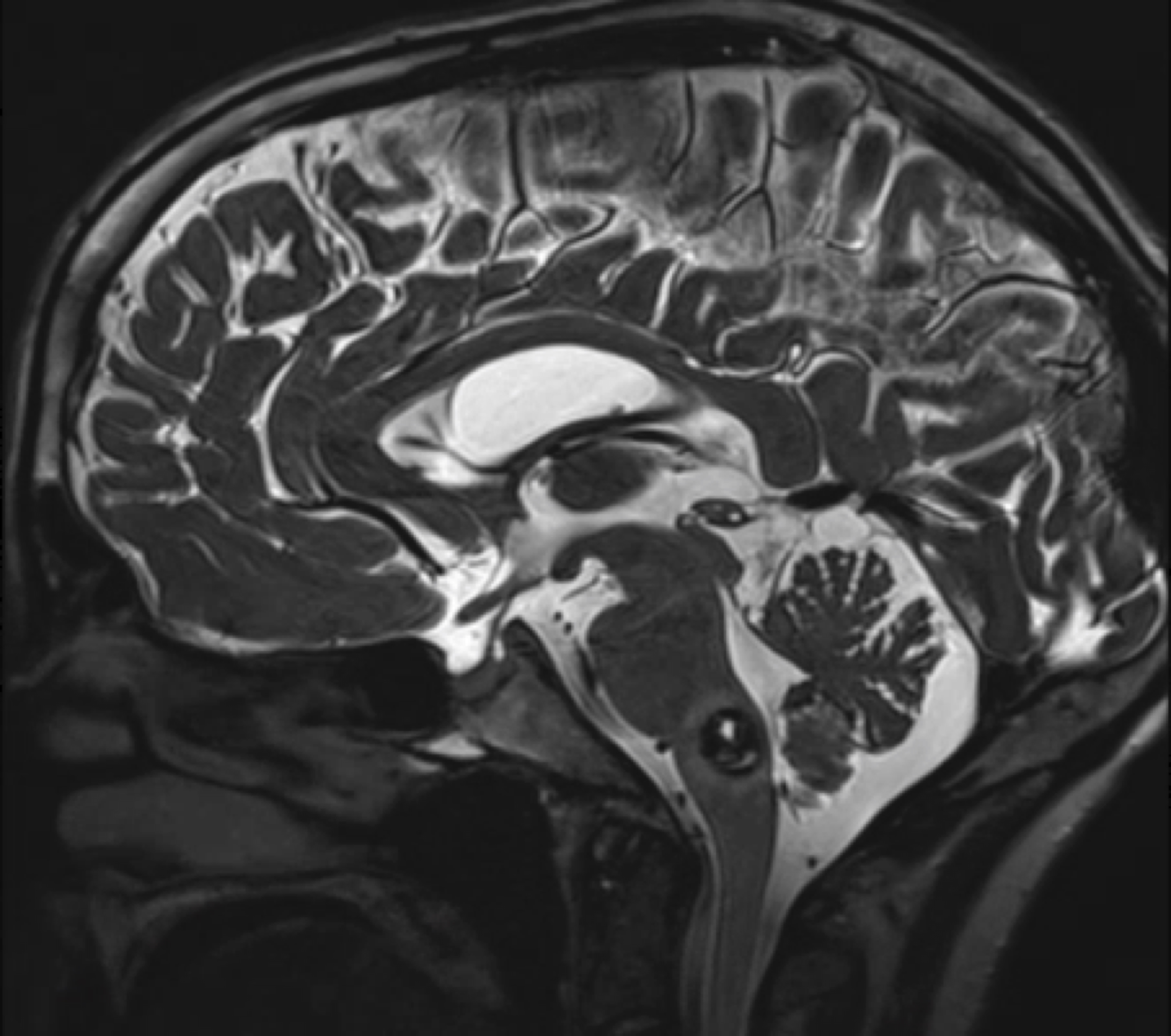

Supplement: Supplementary file 2 — Supplementary file2 (JPG 2857 kb) [file 415_2020_9891_MOESM2_ESM.jpg]

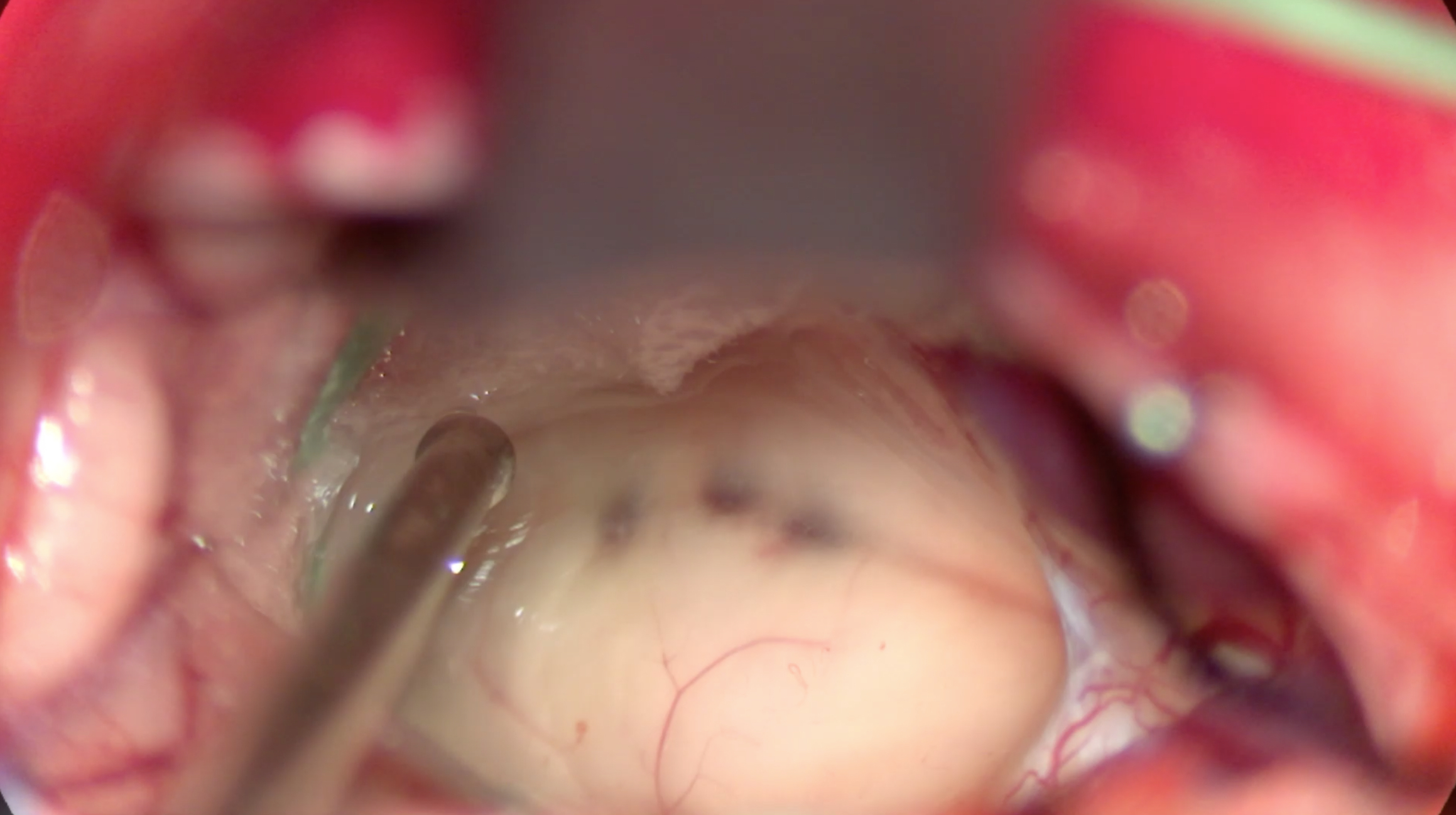

Supplement: Supplementary file 3 — Supplementary file3 Fig. 2 Intraoperative view of the floor of the 4th ventricle. (JPG 1644 kb) [file 415_2020_9891_MOESM3_ESM.jpg]

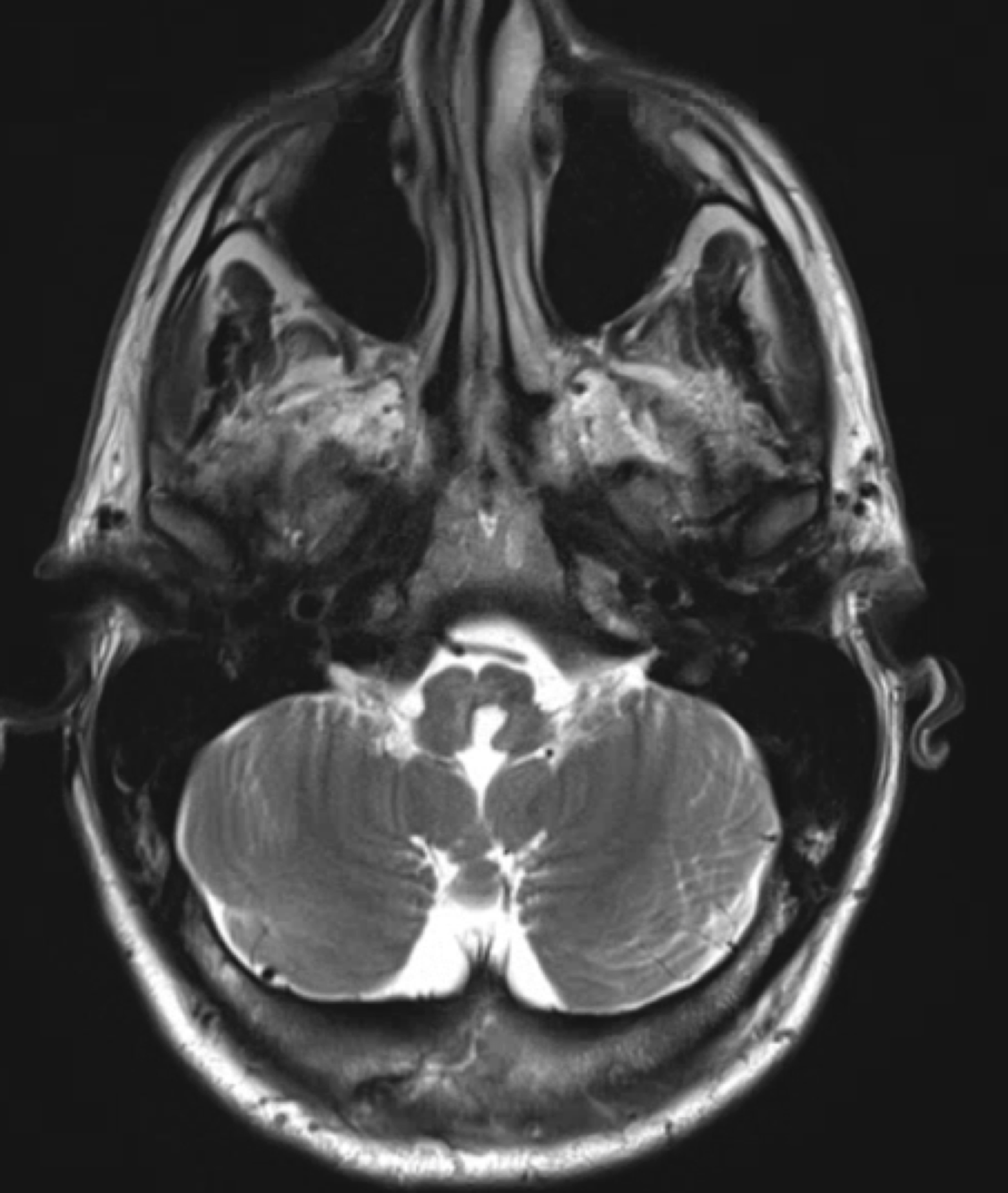

Supplement: Supplementary file 4 — Supplementary file4 Fig. 3 Postoperative 3D T2-weighed high resolution MRI sequences showing complete resection of the cavernoma without radiological complications in axial (A) and sagittal (B) cuts. (JPG 3222 kb) [file 415_2020_9891_MOESM4_ESM.jpg]

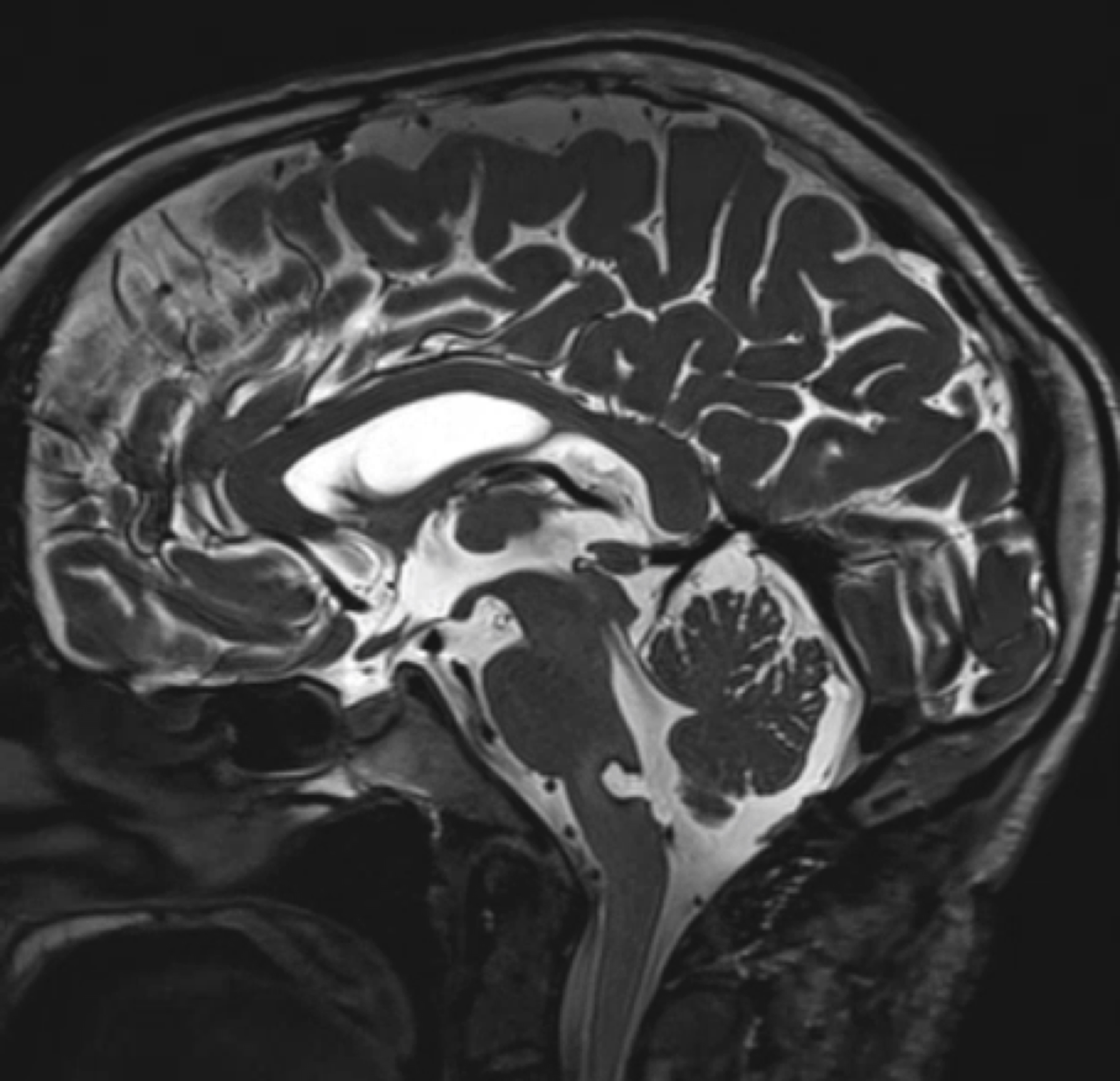

Supplement: Supplementary file 5 — Supplementary file5 (JPG 3014 kb) [file 415_2020_9891_MOESM5_ESM.jpg]
